# Position of the Physician’s Nametag - A Randomized, Blinded Trial

**DOI:** 10.1371/journal.pone.0119042

**Published:** 2015-03-16

**Authors:** Samuel Luca Schmid, Christian Gerber, Mazda Farshad

**Affiliations:** Department of Orthopaedics, University of Zürich, Balgrist University Hospital, Forchstrasse 340, Zürich, Switzerland; Örebro University, SWEDEN

## Abstract

**Background:**

The patient-physician relation begins when the physician introduces himself with name and function. Most institutions request a nametag with name and function to be worn. Although nametags are consequently worn, the optimal position for the nametag is unknown. It was the purpose of this study to identify whether positioning the nametag on the right or the left chest side provides better visibility to the patient.

**Method and Material:**

One hundred volunteers, blinded to the experimental setup, presented for an orthopedic consultation in a standardized manner. The nametag of the physician was randomly positioned on the left chest side and presented to 50 individuals (age 35 years (range 17 to 83)) or the right chest side and then presented to 50 other individuals (35 years (range 16 to 59)). The time of the participant noticing the nametag was documented. Subsequently, the participant was questioned concerning the relevance of a nametag and verbal self-introduction of the physician.

**Results:**

38% of the participants noticed the nametag on the right as opposed to 20% who noticed it if placed on the left upper chest (p = 0.0473). The mean time to detection was 9 (range 1–40) seconds for nametags on the right and 25.2 seconds (range 3 to 49, p = 0.006) on the left. For 87% of the participants, a nametag is expected and important and nearly all participants (96%) expected the physician to introduce himself verbally.

**Conclusion:**

It is expected that a physician wears a nametag and introduce himself verbally at the first encounter. Positioning the nametag on the right chest side results in better and faster visibility.

## Introduction

The patient-physician relation begins at the first encounter. Optimally, the physician introduces himself with name and function. Most institutions request a nametag with name and function to be worn. This is to ensure, that the patient can identify the physician even if the physician does not introduce himself verbally. The importance of a nametag for physicians has been documented previously [1, 2] and efforts were made to enhance the patients' knowledge of the names and roles of the physicians for example by use of face-cards [3]. But, although nametags are well-established and consequently worn in all developed institutions, the correct position of where to wear the nametag is unknown. While in some institutions, the nametag is worn at the left side, in others (e.g. military) it is to be worn at the right side [4, 5]. In most of the hospitals, there is no policy about the position of the nametag. It was the purpose of this study to identify whether positioning the nametag on the right or the left chest side provides better visibility to the patient. Further, we aimed to seek the subjective importance of verbal introduction of the physician to the patient by the first encounter.

## Material and Methods

One hundred volunteers (male to female: 50:50, mean age 35±13, range 16 to 83), blinded to the experimental setup, were included and asked to present for an orthopedic consultation in a standardized manner. All participants were verbally informed and agreed to participate to the experiment. The Ethic Committee of Zurich waived the requirement for approval of this study. Verbal informed consent was obtained from all participants, and participation was voluntary. No medical or personal data were collected, and all data were de-identified during the experiment.

**Fig 1:**
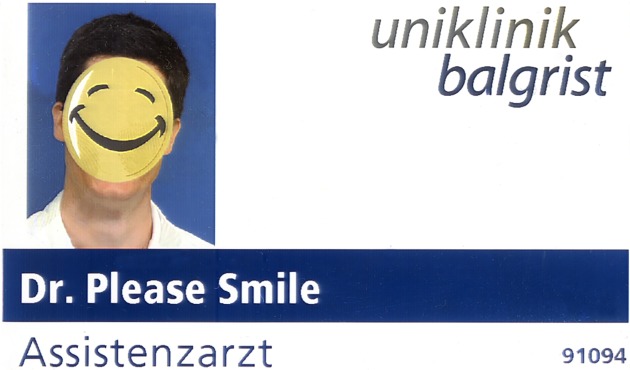
Physicians nametag during consultation.

The participants were blinded to the intervention, namely measurement of time of detection of the nametag. A physician (orthopedic surgeon) randomly a nametag (Fig. 1) either on the left chest side which was then presented to 50 participants (male to female: 25:25, age 35 years (range 17 to 83)) or on the right side which was presented to 50 other participants (male to female: 25:25, 35 years (range 16 to 59)). The same physician was exposed to the participants to avoid confounding of other factors. The physician did consciously not introduce himself by telling his name but used the phrase „*hello*, *why are you presenting at my office*?” and presented the patient his right hand for greeting. For better measurement of the study outcome, namely time until recognition of the nametag, a smiley instead of a photo was used with a text instead of the physicians name: “*Dr*. *Please Smile*” (Fig. 1). An additional independent observer, covered as a medical student, measured the time from entrance of the patient to the room up to patient’s attention to the nametag using an IPhone 4S device. The time and the experiment was stopped, when the patient started to smile while looking at the nametag or tried to spell the doctors name, while entering the room and taking a seat. Otherwise, after 60 seconds, the experiment was terminated. Subsequently, after completion of the experiment, the participant was asked two "yes" or "no" questions. First: “*Is it important that doctors wear a nametag during daily business*” and second: “*Is it important to introduce himself verbally by telling the patient his name and function*”.

### Statistical analyses

The commercial software PRISM (Version 5 for Mac OS X, Graphpad) was used for analysis. Descriptive statistics were used to report mean, standard deviation (SD) and range of data, where adequate. A Kolmogorov-Smirnov test was employed to test for Gaussian distribution before applying either two-sided unpaired student’s t-test or Mann Whitney test for inter-group comparison, respectively. Chi Square test was used to test binary data between the groups. Subgroup analysis was performed to investigate potential gender-dependent differences. A p-value of <0.05 was considered as statistically significant.

## Results

Only 29 of the 100 participants noticed the abnormal nametag. While the nametag was noticed more often (19 of 50, 38%) if worn on the right side, only 20% (10 of 50) noticed it, when it was worn on the left chest side (p = 0.0473). Further, the nametag was noticed much faster if positioned on the right side after 9 seconds (range 1 to 40) if compared with 25.2 seconds (range 3 to 49) if placed on the left chest side (p = 0.006).

The majority (87%) of the participants judged that a nametag was important to wear and nearly all (96%) expected the physician to introduce himself verbally. There was no relevant difference in attitude of the participants in regard to necessity of wearing a nametag or verbal introduction between those who were exposed to a right-sided nametag and those to a left-sided nametag. Latter answered with “yes” in 86% when ask if it is important that a physician wears a nametag and 92% answered with “yes” for importance of verbal introduction. This was similar to the answers of the participants exposed to a right-sided nametag with 88% and 100% respectively.

The mean age of male participants was higher (38±14 years, range 17 to 83) than that of the female participants (32±11 years, range 16 to 53) (p = 0.021). The, attention to the nametag was similar with a detection rate of 26% (13 of 50) for men versus 32% (16 of 50) (p = 0.509) for women. The time for noticing of the nametag was also statistically not different with 15.3s (range 1 to 30) in males versus 14s (range 1 to 49) in females (p = 0.807). The attitude of the male and female patients was nearly equal with 42 males and 45 females considering a nametag as important and 49 male and 47 females emphasizing the necessity of verbal introduction of the physician.

## Discussion

A solid physician-patient communication and relationship is crucial for diagnosis, disease management and control [6–9] and patient satisfaction [10]. The first encounter optimally begins with the physician introducing himself to the patient verbally. Most institutions request that the physician wears a nametag with name and function and while the necessity to do so is well understood and established, it was hitherto unknown whether the nametag should be worn on the left or the right chest side for better visibility. The results of the here presented study clearly demonstrate that positioning the nametag on the right side of the chest results in better and faster detection.

This observation is plausible and could be caused by several behavioral aspects. First, by the first encounter, the patient usually seeks the physician’s eyes first, then searching for his right hand for greeting. Thereby the field of view of the patient runs over the physician’s right side. Second, the physician who reaches out his right arm for salutation presents the right side of his body by doing so.

Nevertheless, anecdotally more physicians (29 of 49 in our institution) are used to place the nametag on the left side of the chest. We believe that this phenomenon is based on the right-handedness (70–90%) [11–13], since it is easier to place the nametag with the right hand on the left chest than on the right side of the chest.

Although the results of this study clearly favor the nametag to be positioned on the right side, limitations of the study need consideration. First, the mean age of the participants was 35 years and the results might not be directly applicable to an older population. Second, the setting of the encounter was an orthopedic consultation and it is plausible, but not yet proven, that the here presented results are also applicable to other settings such consultations of other specialties or to hospitalized patients. Third the study was conducted in a country with a language that is read left-to-right and with the habit to give the right hand for greeting. This might have an effect on the patterns of visual screening of the participants.

## Conclusion

It is expected that a physician wears a nametag and introduces himself (or herself) verbally by the first encounter. Positioning the nametag on the right chest side results in better and faster visibility.
